# Carotid Restenosis: Incidence, Pathophysiology and Therapeutic Options

**DOI:** 10.3390/jpm16020091

**Published:** 2026-02-04

**Authors:** Claudio Bianchini Massoni, Laura Pauletti, Antonio Freyrie

**Affiliations:** 1 Vascular Surgery, Cardio-Thoracic and Vascular Department, University Hospital of Parma, 43126 Parma, Italy; 2 Vascular Surgery, Department of Medicine and Surgery, University of Parma, 43126 Parma, Italy

**Keywords:** carotid artery disease, carotid artery restenosis, in-stent restenosis

## Abstract

Restenosis after carotid endarterectomy and carotid artery stenting remains the main complication after both surgical and endovascular treatment of carotid stenosis, with a 2-year restenosis rate of 6–12%. Complex inflammation processes are the cause of early (<2 years) and late (>2 years) restenosis and principal systemic risk factors are female gender, hypertension, diabetes, dyslipidemia, and smoking. Non-procedural treatment includes lifestyle modifications and best medical therapy. The procedural treatment, considered mostly for symptomatic patients, includes different open and endovascular techniques. The management should be personalized according to patient and plaque characteristics.

## 1. Definition

Carotid restenosis is defined as lumen reduction of an arterial segment that was already treated with carotid endarterectomy (CEA) or carotid stenting (CAS). First description of carotid restenosis was published by Stoney and String in 1976 [1], reporting 29 cases of recurrent disease after CEA.

The grade of lumen reduction and the presence of correlated symptoms define the severity of the condition. At angiographic examination, the restenosis is defined ≥50% and ≥80% according to the North American Symptomatic Carotid Endarterectomy Trial (NASCET) [2]. However, the majority of clinical trials consider 70% the threshold for high-grade restenosis [3,4,5]. The restenotic lesion is asymptomatic in the majority of cases, but can cause neurological cerebral symptoms (transient ischemic attack [TIA] or stroke). The patients with post-CEA or post-CAS restenosis are burdened by a four-fold increase in the risk of stroke compared with patients with no restenosis [6].

The restenosis should be discerned from recurrent stenosis. This term states an arterial narrowing identified after 4–6 weeks from the intervention and it is suggested for residual atherosclerotic disease [7].

## 2. Epidemiology

The incidence of restenosis after CEA or CAS is highly variable depending on restenosis definition, technique of first treatment, diagnostic methods, and time from intervention. After CEA, a historical publication of 1982 [8] reports high-grade restenosis in 19% of cases at 16 months. A huge monocentric experience published in 2005 reports >70% restenosis after patch-CEA in 8.5% at 5 years, including early and late restenosis in 3.8% and 5.8%, respectively [9]. A recent Cochrane analysis reports long-term >50% restenosis after CEA of 8.7%, with a large difference between patch-plasty CEA and primary-closure CEA (4.3% vs. 13.8%, respectively) [10]. Restenosis is also a possible complication after CAS. According to the Carotid Revascularization Endarterectomy versus Stenting Trial (CREST), the 70% restenosis after CAS is comparable to CEA restenosis during 2 post-operative years (CAS 6% vs. CEA 6.3% [6]). The Endarterectomy versus Stenting in Patients with Symptomatic Severe Carotid Stenosis (EVA-3S) [5] trial underlines that the rate of ≥50% restenosis after 3 years is higher after stenting than surgery (CAS 12.5% vs. CEA 5%, *p* = 0.02); however, ≥70% restenosis is lower in both groups without statistical significance differences (CAS 3.3% vs. CEA 2.8%). On the other side, the SPACE trial [4] finds difference in terms of ≥70% restenosis rate at 2 years after CAS and CEA (11.1% vs. 4.6%, respectively). The restenosis rates of these three trials are summarized in Table 1.

## 3. Pathophysiology

On the basis of time occurrence, the restenosis could be classified into early (<2 years) or late (>2 years) [11,12]. These two events seem to be completely different entities, resulting from parallel processes and leading to distinct patient risks. Early restenosis is induced by an excessive reaction to the arterial trauma of the previous treatment. Intimal hyperplasia is the main process responsible for early restenosis, consisting of intimal thickening following endothelial damage both after endoarteretomy [13] and stenting [14]. The wall microtrauma leads to the recruitment and activation of monocytes and macrophages, production of inflammatory mediators (grow factors, chemokines, cytokines, vascular factors, adhesion molecules and free radicals), and migration and proliferation of smooth muscle cells to the intima. The neo-plaque induced by intimal hyperplasia is fibrous and fairly stable, with low tendency to embolization [12]. On the other hand, late restenosis is considered to be the consequence of progression of atherosclerotic processes [12].

The inflammatory processes involved in early and late restenosis are complex and not fully understood. Several studies try to find inflammation biomarkers associated with carotid plaque progression and restenosis [15,16]. A large population-based study (4334 patients) proved that the inflammation is a risk of plaque progression, independent dyslipidemia, and other risk factors; moreover, the levels of circulating interleukin-6 (IL-6) are in an independent linear relationship with carotid plaque progression at 5 years [15]. Scalise et al. [16] considered 68 CEA and 32 CAS patients and found preintervention neutrophils-to-lymphocytes ratio and systemic immune response index to be independent predictors of >50% restenosis at 12-month follow-up.

## 4. Risk Factors

Due to their different pathophysiology, the risk factors of early and late carotid restenosis should be different. However, this difference in time of occurrence is not always considered when dealing with carotid restenosis. The predictors of restenosis after CEA or CAS are usually classified in systemic factors and technical predictors (Table 2). Systemic risk factors for 2-year restenosis (both post-CEA and post-CAS) are female gender, hypertension, diabetes, and dyslipidemia [6], while smoking is associated with restenosis after CAS and not after CEA [6,17]. The International Carotid Stenting Study (ICSS), comparing CEA and CAS, also detected other negative factors for ≥50% restenosis to be older age, history of angina, and higher total serum cholesterol [18].

Considering technical aspects, CEA with patch closure leads to lower ≥50 and ≥70% restenosis than primary closure [10,19,20], while no statistical differences were found between patch closure and eversion technique [21,22]. The intraoperative use of shunt increases restenosis risk [23]. Considering CAS, second-generation stents (dual-layer stents) seem to improve short- and long-term outcomes [24,25], including a reduction in in-stent restenosis [25].

Some authors hypothesize that features of carotid plaque at ultrasonography influence the rate of restenosis. Liapis et al. [26] considered 338 carotid axes, finding a higher rate of restenosis after CEA for echolucent compared to echogenic plaques during follow-up (mean 63 months; echolucent 22% vs. echogenic 5%; *p* = 0.005). In another study [23], which measured plaque grayscale median at ultrasound after CEA, echolucent carotid plaques had a higher 1-year restenosis rate than echogenic plaques. Even calcification of plaque seems to have an effect on the restenosis. In Katano’s experiment [27], 6-month restenosis is more frequent in high-calcified plaque after both CAS and CEA (18.8% and 20.0%, respectively).

Some serum molecules are correlated with restenosis rate after CEA. High levels of mannose-binding lectin and C-reactive protein, and low levels of homocysteine, apolipoprotein J and vitamin C are related with a higher rate of restenosis during follow-up after CEA [13].

Statin is a carotid plaque drug therapy used to decrease stroke and death rates. However, statin therapy seems to have no influence on restenosis rate after CEA [28]. From a recent meta-analysis, Cilostazol is associated with less carotid stent restenosis [29].

**Table 2:** Classification of the main risk factors for carotid restenosis according to previous treatment.

Type of Risk Factors	Risk Factors	Previous Treatment	References
Systemic	Female gender	CEA CAS	[6]
	Hypertension	CEA CAS	[6]
	Diabetes	CEA CAS	[6]
	Dyslipidemia	CEA CAS	[6]
	Smoking	CAS	[6,17]
	Older age	CEA CAS	[18]
	History of angina	CEA CAS	[18]
Technical	Primary closure	CEA	[10,19,20]
	Intraoperative shunt	CEA	[23]
	First-generation stent	CAS	[25]

CEA: carotid endarterectomy; CAS: carotid artery stenting.

## 5. Diagnosis

The examination used to detect a carotid restenosis is duplex ultrasound (DUS). The ultra-sonographic criteria for diagnosis of restenosis is controversial. It is not clear if peak systolic velocity (PSV) is the same for primary atherosclerotic disease. After CEA, AbuRama et al. [12] define that >50% and >70% restenosis (NASCET criteria) have a PSV ≥213 cm/s and ≥274 cm/s, respectively. After CAS, stent induces an alteration of stented-artery compliance, increasing in-stent PSV. Different studies compare DUS, computed tomography angiography (CTA) and digital subtraction angiography (DSA) after stenting to obtain PSV and internal/common PSV (internal carotid artery [ICA]/common carotid artery [CCA]) ratio thresholds [30,31]. Stanziale et al. [31] suggest PSV ≥ 225 cm/s and ICA/CCA ratio ≥ 2.5 for ≥50% in-stent restenosis and PSV ≥ 350 cm/s and ICA/CCA ratio ≥ 4.75 for ≥70% in-stent restenosis. Lal et al. [30] consider in-stent restenosis ≥ 50% with PSV ≥ 220 cm/s and ICA/CCA ratio ≥ 2.7, and in-stent restenosis ≥ 80% with PSV ≥ 340 cm/s and ICA/CCA ratio ≥ 4.15.

CTA is a second-level examination that permits the analysis of CCA and ICA (including intracranial ICA), but does not allow hemodynamic evaluation, and involves ionizing radiation and nephrotoxic contrast agent injection. In addition, in the presence of severe calcified lesions, it may overestimate the grade of plaque stenosis [32].

Magnetic resonance (MR) can obtain high-quality images if performed by expert radiologists. The major pro is the absence of ionizing radiation, but high costs, the not immediate availability and long acquisition times reduce its application [32].

## 6. Best Medical Therapy in Carotid Restenosis: A Targeted Strategy

Best Medical Therapy (BMT) in patients with carotid restenosis plays a central and tailored role, as we already highlighted; however, the mechanisms of restenosis differ substantially from those of de novo atherosclerotic carotid disease. Whereas primary carotid stenosis is predominantly driven by plaque rupture and thrombosis, restenosis is largely a consequence of intimal hyperplasia (early restenosis < 2 years) or recurrent atherosclerosis (late restenosis > 2–3 years). Therefore, medical management must be adapted to address both thromboembolic risk and biological drivers of vascular remodeling. Thus, BMT is the cornerstone of management for both symptomatic and asymptomatic carotid restenosis. While revascularization is indicated in selected cases of restenosis, BMT remains a critical component across all clinical presentations and is frequently the sole treatment option in asymptomatic or high-surgical-risk individuals.

### 6.1. Antiplatelet Therapy in Restenosis

Long-term single antiplatelet therapy with low-dose aspirin (75–325 mg daily) is recommended for all patients with >50% carotid stenosis (class IIa, level C) mainly for the prevention of late myocardial infarction and other cardiovascular events [7]. Specifically, a patient with restenosis should already be on life-long antiplatelet therapy from the time the revascularization (surgical or endovascular) was first performed.

In cases of post-CEA restenosis, long-term single antiplatelet therapy with aspirin remains standard, aiming to prevent thromboembolic events from plaque destabilization. However, restenotic lesions tend to be fibrotic and less lipid-rich than native atherosclerotic plaques, and the actual embolic potential may be lower in purely fibrotic restenosis [33]. For this reason, escalation to dual antiplatelet therapy (DAPT) is not routinely indicated unless intervention is planned or the patient presents with symptoms suggestive of a thromboembolic origin.

In post-CAS restenosis, single antiplatelet therapy is sufficient in cases of asymptomatic restenosis with no indication for reintervention. On the other hand, DAPT is mandatory for at least one month (and up to three) following any in-stent angioplasty or re-stenting intervention to prevent thromboembolic events. Prolonged DAPT beyond 1–3 months is not recommended in the absence of new ischemic events [7].

Importantly, in-stent restenosis may present a higher thrombogenic potential due to endothelial dysfunction and strut-related flow alteration, especially in under-expanded stents or those with edge restenosis [34].

As previously anticipated, in cases of symptomatic restenosis, either post-CEA or post-CAS, upgrading to DAPT is necessary and should be undertaken peri-procedurally. Clopidogrel, Dipyridamol or ticagrelor may be considered in aspirin-intolerant patients as monotherapy [7].

### 6.2. Lipid-Lowering Therapy: Focus on Neointimal Hyperplasia and Atheroprotection

Although neointimal hyperplasia, the predominant mechanism of early restenosis, is not directly atherosclerotic, evidence supports a role for statins in modulating vascular smooth muscle proliferation and improving endothelial function, both of which are implicated in neointimal hyperplasia development. Statins also have a known pleiotropic effect on inflammation, oxidative stress, and matrix remodeling, making them uniquely suited for late-restenosis prevention [26].

The LDL target remains <70 mg/dL, and a ≥50% reduction from baseline is recommended. Kim et al. [35] show that CAS patients with an LDL level ≥ 70 mg/dL during follow-up have a higher risk of restenosis compared with patients with an LDL < 70 mg/dL. In high-risk patients or statin-intolerant patients, intensification with ezetimibe or PCSK9 inhibitors is advised (class IIa, level C) [7].

### 6.3. Blood Pressure and Glycemic Control

Given the vascular remodeling and endothelial dysfunction involved in restenosis, meticulous control of hypertension and diabetes is essential. The hemodynamic stress from elevated systolic blood pressure, particularly in the presence of contralateral occlusion or incomplete circle of Willis, may contribute to plaque instability and accelerate restenotic progression.

- Blood pressure target: <140/90 mmHg (general), <130/80 mmHg in high-risk patients.
- HbA1c target: <7.0% in most; tighter control in younger, low-risk patients.

### 6.4. Smoking Cessation and Lifestyle Measures

For patients with asymptomatic and symptomatic carotid disease, behavioral counseling to promote healthy diet, smoking cessation and physical activity is recommended by ESVS guidelines (class I, level B) [7]. Their role in reducing cardiovascular morbidity is well-established and remains applicable in the context of restenosis.

Smoking is a strong, modifiable predictor of restenosis. Nicotine induces vasoconstriction, promotes smooth muscle cell proliferation, and worsens endothelial repair mechanisms. Its impact on in-stent restenosis is particularly pronounced. All patients with restenosis—regardless of symptom status—should receive structured cessation support.

Dietary counseling, physical activity (defined as at least 150 min/week of moderate aerobic activity), and weight control/weight loss in obese patients are all key non-pharmacological interventions (endorsed by the European Society for Vascular Surgery [ESVS] [7]). Healthy diets and physical activity reduce the risk of cardiovascular events at 24 months with a relative risk reduction of 0.80, and obesity is associated with stroke (relative risk increase: 1.64) [7].

## 7. Indications for Reintervention

The decision to reintervene after carotid restenosis, whether occurring after CEA or CAS, must consider multiple variables: whether the restenosis is symptomatic or asymptomatic, the degree and progression of stenosis, comorbidities, and prior treatment modality. A consensus on the treatment of choice for carotid restenosis remains unclear based on currently available literature, nonetheless it must take into consideration the patient risk status as well as the proficiency of the center where the procedure is taking place.

According the 2023 ESVS guidelines and the 2022 Italian Society of Vascular and Endovascular Surgery guidelines, the intervention is not routinely indicated, especially in asymptomatic cases, while BMT must be always optimized in order to reduce the risk of thromboembolic events as well as further progression of the restenosis [7,36].

### 7.1. Asymptomatic Restenosis

In cases of asymptomatic restenosis, BMT is the primary therapy used in order to prevent the progression of stenosis, avoid late thromboembolic events, as well as reduce cardiovascular mortality. BMT may defer or even eliminate the need for revascularization, particularly in elderly, frail, or comorbid patients where the procedural risk outweighs potential benefits.

According ESVS guidelines [7], reintervention is recommended for asymptomatic restenosis after CEA in cases of stenosis between 70% and 99% following multidisciplinary team review (class IIb, level A). After CAS, for patients who develop an asymptomatic restenosis >70% medical management is recommended (class I, level A).

Factors that may suggest the need for intervention are: signs of progression, contralateral carotid occlusion or poor intracranial collateral flow, high-risk plaque features (e.g., intraplaque hemorrhage, ulceration, mobile thrombus) at imaging findings, and young patient who is otherwise at low surgical/anesthetic risk [7,37].

### 7.2. Symptomatic Restenosis

Revascularization is strongly recommended in patients presenting with 50–99% symptomatic restenosis (TIA, amaurosis fugax, or stroke referable to the restenotic side) if other causes of neurological symptoms (e.g., cardioembolism) have been excluded. The 2023 ESVS guidelines (class I, level B) recommend revascularization as the preferred strategy when the benefits of stroke prevention outweigh the procedural risks [7].

Nonetheless, BMT must be optimized before and after intervention, as restenosis—particularly when due to neointimal hyperplasia—may recur even after retreatment. Medical therapy is essential to stabilize the vascular endothelium and reduce long-term atherothrombotic risk, thus minimizing the risk of recurrence.

Also, the Guidelines of the Italian Society of Vascular and Endovascular Surgery published in 2022 suggest the treatment of post-CEA or post-CAS moderate or severe restenosis only in cases of related symptoms [36].

## 8. Choice of Treatment

Selecting the most appropriate revascularization technique for carotid restenosis requires careful integration of patient-specific risk factors, anatomical and technical considerations, prior treatment (CEA vs. CAS), plaque morphology and timing of restenosis, and availability and experience with newer techniques such as Transcarotid Artery Revascularization (TCAR). A careful assessment of patient condition and comorbidities, symptoms, and plaque characteristics at duplex and/or CTA are essential to propose the lower-risk management for each patient.

As suggested by the 2023 ESVS guidelines [7], the revascularization technique should be chosen after a multidisciplinary team review, including local surgeon and interventionist preference and patient choice (class I, level C). Type and outcome of each procedure are summarized in Table 3.

**Table 3:** Types of reintervention technique with main outcome.

	Reintervention	Outcome	References
Retreatment post-CEA	CAS	2-year restenosis recurrence: 5.2%	[38]
	Redo-CEA	5-year restenosis: 4.4%; cranial nerve injury: 5–7%	[39,40]
Retreatment post-CAS	POBA	2-year restenosis: 20–40%	[41]
	Stent-in-stent CAS	Long-term in-stent restenosis: 8.2%	[41]
	DCB angioplasty	30-month recurrent stenosis: 15.6%	[42]
	CBA	Recurrent severe in-stent restenosis: 11.1% (median follow-up: 21 months, range 9–110)	[43]
	Open conversion (CEA, carotid bypass)	Mid-term restenosis comparable to re-stenting	[34]

CEA: carotid endarterectomy; CAS: carotid artery stenting; POBA: plain old balloon angioplasty; DCB: drug-coated balloon; CBA: cutting balloon angioplasty.

### 8.1. Retreatment Post-CEA

Most post-CEA restenoses are asymptomatic, in which case BMT and surveillance are usually recommended [7,36]. The need for reintervention is dictated by symptom status, stenosis severity, and imaging progression, with reintervention being reserved for symptomatic or high-grade lesions [38,39,41].

Treatment options for post-CEA restenosis are carotid artery stenting (CAS), redo-endarterectomy (Redo-CEA) or trans-carotid artery revascularization (TCAR) [7,39,40].

#### 8.1.1. Carotid Artery Stenting (CAS)

**Figure 1:**
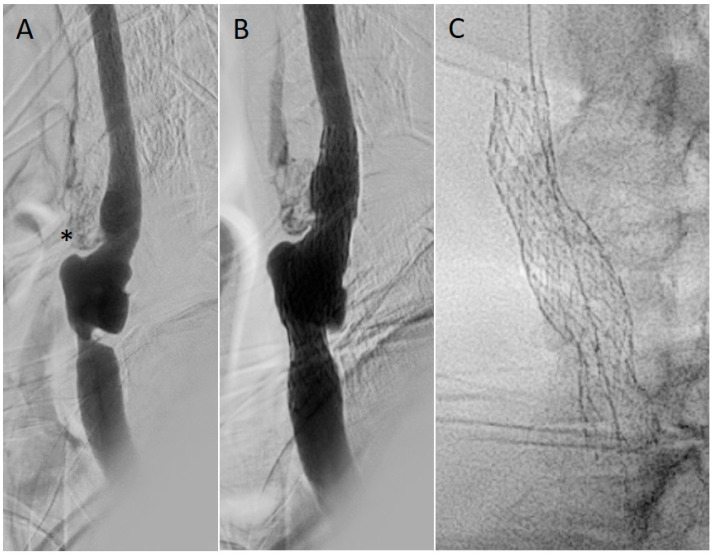
Treatment of an asymptomatic >80% restenotic lesion of the common carotid artery after previous carotid endarterectomy with Dacron patch plasty. (**A**) Angiography (* occlusion of external carotid artery); (**B**) completion angiography after dual-layer stent deployment; (**C**) radiographic aspect of dual-layer stent.

**Figure 2:**
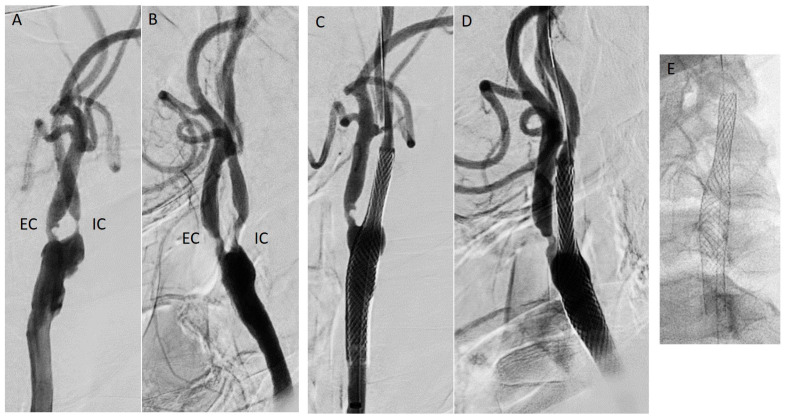
Treatment of a subtotal symptomatic restenotic lesion of the left internal carotid artery after previous carotid endarterectomy with patch plasty (bovine pericardium). (**A**,**B**) Orthogonal angiographies (EC: external carotid; IC: internal carotid); (**C**,**D**): completion angiography after closed-cell stent deployment; (**E**): radiographic aspect of closed-cell stent.

CAS (Figure 1 and Figure 2) consists of the deployment of a stent at the site of the restenosis and is often preferred as a first-line reintervention, especially in cases of high-risk patients or hostile surgical fields where re-operating may put patients at increased risk of cranial nerve injury and hematoma secondary to loss of tissue planes [39]. Restenotic lesions after carotid interventions most commonly demonstrate a concentric, fibrotic neointimal pattern, particularly in case of early restenosis (<2 years), and are therefore less eccentric and ulcerated than de novo atheromatous plaques. This concentric, smooth, fibromuscular hyperplasia phenotype has implications for endovascular treatment because it tends to respond to controlled mechanical plaque modification (careful pre-dilation) rather than aggressive debulking, thus a balloon angioplasty is usually performed before stenting [33]. Dual-layer or mesh stents (e.g., CGuard, Roadsaver/Casper) are sometimes preferred for improved plaque coverage and reduced embolic risk over bare metal stents. Given the potential for plaque prolapse and embolization, second-generation dual-layer (micromesh/mesh) stents have been developed to increase plaque coverage and reduce embolic showering during and after deployment. When selecting a stent for post-CEA restenosis, micromesh stents are therefore often preferred in lesions with significant plaque burden or where embolic protection is a priority [44,45]. Patch-related restenosis (from prior Dacron, vein, or bovine pericardium patch) may have a more focal morphology, with a well-defined landing zone, facilitating safe stent deployment. CAS is less invasive than redo surgery, avoids scar dissection, and correlates with lower cranial nerve injury rates as well as granting shorter hospital stay [40]. On the downside, this technique has a higher potential for in-stent restenosis, with long-term durability being somewhat less robust than redo-CEA in some series. Meta-analyses [34,40] show comparable stroke/death risk between redo-CAS and redo-CEA, with lower cranial nerve injury risk for CAS. Dorigo et al. [38] reported no difference in perioperative outcomes, while demonstrating how restenosis recurrence is slightly higher after CAS (5.2% vs. 3.7%) at 2 years. Further limitations of this technique, such as the risk of embolic events still remaining higher in case of friable or ulcerated lesions, can be reduced by proximal or distal embolic protection devices (EPDs) or TCAR. Moreover, in the case of anatomical limitations, such as hostile aortic arch or poor distal landing zone, CAS may be precluded. CAS should always be performed within the framework of aggressive medical optimization, specifically, DAPT needs to be started at least 5 days before the procedure and continued for the 3 following months [7], which can complicate management in patients with bleeding risk. Statins should be implemented if not already administered and adherence to BMT is essential, as recurrent restenosis and late thromboembolic events are strongly associated with suboptimal lipid and blood pressure control [7,46]). The ESVS guidelines 2023 [7] support the use of CAS as the preferred option for restenosis after CEA in patients at high surgical risk. Surveillance is recommended through duplex ultrasound at 1, 6, and 12 months, then annually.

#### 8.1.2. Redo-Carotid Endarterectomy (Redo-CEA)

Redo-carotid endarterectomy (Redo-CEA) represents the traditional surgical approach for managing carotid restenosis after primary endarterectomy [7,37]. Before the advent of CAS, redo-CEA was the mainstay for symptomatic restenosis, particularly when restenosis resulted from technical failure or progressive atherosclerosis rather than myointimal hyperplasia. Although the technique offers definitive plaque removal and allows direct inspection of the arterial reconstruction, it is technically demanding due to fibrotic scarring, adhesions, and the proximity of cranial nerves and patch material [40]. Therefore, patient selection and surgical expertise are crucial to minimizing morbidity. It is therefore a therapeutic option that should be reserved for selected low-risk patients and cases where stenting is contraindicated, such as heavily calcified lesions, narrow distal landing zones, hostile aortic arch or prior neck radiation precluding safe stent access [38]. Since it is technically more challenging than first-line CEA due to scar tissue, adhesions and distorted anatomy and is burdened with higher risk of cranial nerve injury (up to 5–7% vs. <3% in primary CEA), redo surgery should ideally be performed in high-volume centers with vascular surgical expertise given the increased technical difficulty and complication potential [39]. Redo-CEA may involve: re-exposure of the carotid bifurcation through the previous incision, requiring careful dissection of scar tissue and protection of cranial nerves (VII, X, XII); endarterectomy extension into the ICA beyond the previous endpoint to ensure a smooth distal transition; patch angioplasty, using bovine pericardium, Dacron, or polytetrafluoroethylene (PTFE), to prevent re-narrowing; and lastly, bypass or interposition grafting when the arterial wall is severely damaged or fibrosis is extensive. In selected patients with a hostile neck, a retro-jugular approach or limited distal dissection may be considered to reduce morbidity [7,37,38].

### 8.2. Retreatment Post-CAS

Treatment options for post-CAS restenosis are either endovascular or surgical. Due to the absence of treatment guidelines for in-stent restenosis (ISR) after CAS, the selection of treatment options varies depending on the experience of treating physicians and centers [7,37]. Endovascular approaches (balloon angioplasty with various types of balloons or adjunctive stenting) or surgical interventions (CEA with stent explantation, carotid artery bypass, or interposition graft) can be considered as viable options for ISR [47]. In particular, the following procedures are most commonly undertaken:

#### 8.2.1. Plain Old Balloon Angioplasty (POBA)

POBA uses conventional balloon dilation to restore lumen patency within a restenotic carotid stent. It is technically straightforward and avoids the need for further stent implantation (which increases metal burden and complicates future reinterventions) since its mechanism of action is intimal compression and stretching. On the other hand, it does not address the biological drivers of neointimal hyperplasia. The procedure’s technical success is high (reported >90%), with excellent periprocedural safety: stroke and death rates after POBA are low (<3% in most reports). The main advantages of this technique are the relative technical simplicity, the fact that is widely available, and that it avoids additional stent layers. It can be used as an initial treatment or in patients with limited anatomy/surgical options and may as well serve as a bridge therapy in patients at high surgical or procedural risk. Its main limitation is a high recurrence rate (20–40% at 1–2 years in several observational series) due to elastic recoil and continued neointimal proliferation due to the absence of anti-proliferative therapy (unlike drug-coated balloon [DCB]) [41]. Moreover, repeated POBA sessions may be required, increasing overall procedural burden. Guo Z. et al. [34] reported how POBA alone has the highest recurrence rates among ISR treatment options. The same ESVS 2023 guidelines note that POBA can be considered as a feasible and safe treatment option, but outcomes are inferior to alternative approaches such as DCB or re-stenting [7].

#### 8.2.2. Re-Stenting (Stent-in-Stent CAS)

**Figure 3:**
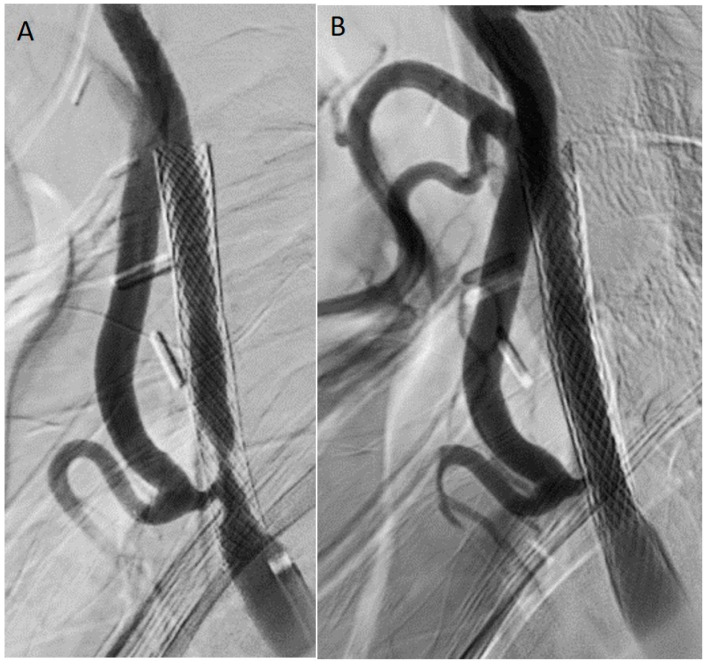
Treatment of an in-stent restenotic lesion for asymptomatic internal carotid artery severe stenosis (peak systolic velocity >400 cm/s). (**A**) Angiography; (**B**) completion angiography after closed-cell stent deployment (stent-in-stent).

Re-stenting (Figure 3) consists on the deployment of an additional stent within the existing stent to provide a mechanical scaffold to treat recoil, seal dissection, or recurrent ISR. Registry and meta-analytic data show reasonable technical success and symptom control. Comparative studies (meta-analyses) indicate similar short-term outcomes between re-stenting and surgical conversion in selected cohorts [34,41]. When considering possible disadvantages associated with this technique, it is worth noting that the metal burden may increase chronic inflammation and risk for further ISR over time; moreover, it makes future surgical stent explantation more difficult. The stent overlap may also affect the flow/hemodynamic. Thus, this technique must be considered when a mechanical issue (recoil/dissection, under-expansion) is the dominant problem or when first-line treatments with DCB/cutting balloon angioplasty (CBA) fail or are contraindicated [34].

#### 8.2.3. Drug-Coated Balloon (DCB) Angioplasty

DCB is an emerging option for focal ISR [48]. It consists of balloon angioplasty with local delivery of an antiproliferative drug (e.g., paclitaxel) to the vessel wall without the need to place additional metal. It combines mechanical dilatation with local pharmacologic inhibition of smooth muscle cell proliferation, thus addressing one of the biological drivers of ISR [7]. Evidence of its efficacy in carotid ISR is growing but still limited to case series and small retrospective cohorts; early-/mid-term results show lower restenosis rates compared with POBA alone [42,48]. The choice of using a DCB seems to be increasingly favored for focal ISR and is ideal when the operator wishes to avoid further stenting. It is even considered as a first-line endovascular therapy in centers with experience [42,48,49].

#### 8.2.4. Cutting Balloon Angioplasty (CBA)

CBA can be considered the technique of choice in cases of intra-stent restenosis, particularly in cases with high calcification. CBA can achieve a wider gain in luminal diameter compared to POBA while posing a lower risk of stent overexpansion due to the nature of the technology [50]. First, CBA requires fewer attempts at balloon angioplasty when compared with POBA. Second, the rate of additional stent placement tends to be lower. Third, CBA shows a lower frequency of balloon slippage due to microblades (atherotomes) anchoring the balloon to the plaque during inflation, which subsequently reduces the risk of arterial dissection near the stent margins. Fourth, the microblades on the surface of the cutting balloon can surgically incise the neointimal tissues or atherosclerotic plaque within the stent and extrude fragmented tissues or plaque out of the stent. Lee et al. [43] showed how during a period spanning from 2012 to 2021, a total of 20 severe ISRs in 18 patients (including 4 symptomatic) following CAS underwent CBA (median time interval between initial CAS and ISR detection was 390 days (interquartile range 324–666 days), and median follow-up was 21 months, (range 9 months–9 years)). Out of the 18 patients receiving CBA, 16 (88.9%) did not require additional stenting and 16 (88.9%) did not experience recurrent ISR during the follow-up period. Two patients were successfully treated with CBA and additional stenting. No peri-procedural complications were observed in any case.

Despite several advantages of a CBA over POBA, it is important to note that CBA carries the risk of vessel injury or arterial perforation due to the microblades. As an answer to this, it has been proposed that CBA can be safely performed for ISR after CAS, as the implanted stent acts as a protective barrier against potential microblade-induced vessel wall injury. Hence, a CBA could be safely inflated up to the stent diameter. Zhou et al. [51] reported cutting balloon angioplasty as safe, with better short-term luminal gain compared to conventional angioplasty in ISR, though long-term durability was not superior. Guo et al. [34] highlighted CBA as an “effective but not definitive” option, with restenosis recurrence still common. The 2023 ESVS guidelines [7] do not provide a strong recommendation for CBA due to the lack of high-level evidence, but acknowledge its use as a bailout or adjunctive strategy in ISR when conventional angioplasty is insufficient [7].

#### 8.2.5. Open Conversion (CEA, Carotid Bypass)

Even if endovascular reintervention (re-stenting, CBA, DCB) is the mainstay for ISR after CAS, open surgical conversion must be considered in diffuse, high-grade, or recurrent restenosis not amenable to endovascular therapy, or when anatomy/device failure complicates further intervention. Moreover, surgery may also be chosen when patients remain symptomatic despite optimal medical and endovascular reintervention. The ESVS 2023 guidelines [7] recommend that open conversion (CEA ± stent explantation) may be considered in patients with symptomatic, high-grade ISR after CAS if endovascular strategies are unsuitable or have failed [7,34,39].

- CEA with stent explantation is technically demanding and reserved for select low-risk patients. One key limitation to treating stent restenosis with CEA is that the vast majority of the CAS patients had an underlying condition that made them at a high risk for primary CEA. These same factors, such as high cervical lesions and previous radiation, must be accounted for in the setting of restenosis as well. CEA after CAS can and is safely performed as treatment for symptomatic (any degree) and asymptomatic >80% restenosis [7,31,39].
- Carotid bypass (extra anatomic or in situ reconstruction) is rarely performed, typically left for unsalvageable arterial segments or heavily calcified vessels where endarterectomy is unsafe. Technical options include common-to-internal carotid bypass with vein or prosthetic graft.

### 8.3. Transcarotid Artery Revascularization (TCAR)

TCAR is a technology which involves the exposure of the proximal common carotid artery above the clavicle so that an arterial sheath can be positioned immediately proximal to the target lesion. Before traversal of the lesion with a guidewire, the proximal common carotid artery is clamped with an external filter circuit (ENROUTE Neuroprotection System—Silk Road) connecting to the femoral vein, which results in cerebral flow reversal. In this way, dislodgement of plaque from the target lesion is externalized through the sheath, filtered, and returned to the venous circulation via a previously placed femoral vein sheath, reducing the risk of embolic stroke. Additionally, the aortic arch is not manipulated during device positioning, further eliminating the risk of embolic arch debris [52].

Although TCAR seems to not replace trans-femoral CAS or CEA [53], a consensus on the treatment of carotid restenosis remains unclear based on currently available literature. TCAR can be effectively and safely performed in patients presenting with ipsilateral restenosis after CEA, reducing the risk of 30-day TIA with comparable stroke, death and myocardial infarction in comparison with trans-femoral CAS [54]. Carotid restenotic lesions seem to be a risk factor for restenosis after TCAR [55]. Wang et al. [56], when considering a single-center experience of 55 patients with carotid restenosis (47 after CEA and 8 after trans-femoral CAS), observed no ipsilateral strokes or deaths, one myocardial infarction (1.8%), and no reinterventions or stent thromboses.

## 9. Conclusions

Restenosis remains the main complication after CEA and CAS. Best medical treatment is mandatory, but none of the procedural treatments have such superiority to become the unique restenosis approach. The management has to be personalized based on previous treatment (CEA or CAS), the patient’s conditions, symptoms, imaging, surgeon experience, and patient’s preference.

## Figures and Tables

**Table 1:** Summary of restenosis rate in three trials.

Study	Restenosis Definition	Restenosis/Occlusion Rate			References
		At time	CEA	CAS	
CREST	70% restenosis or occlusion	4 years	6.2%	6.7%	[6]
SPACE	>70% recurrent stenosis	2 years	4.6%	11.1%	[4]
EVA-3S	≥50% stenosis or occlusion	3 years	5%	12.5%	[5]
	≥70% stenosis or occlusion		2.8%	3.3%	

CREST: Carotid Revascularization Endarterectomy versus Stenting Trial; SPACE: Stent-Protected Angioplasty versus Carotid Endarterectomy; EVA-3S: Endarterectomy vs. Angioplasty in Patients with Symptomatic Severe Carotid Stenosis; CEA: carotid endarterectomy; CAS: carotid artery stenting.

## Data Availability

No new data were created or analyzed in this study.

## Abbreviations

The following abbreviations are used in this manuscript:

CEA	Carotid endarterectomy
CAS	Carotid artery stenting
NASCET	North American Symptomatic Carotid Endarterectomy Trial
TIA	Transient ischemic attack
CREST	Carotid Revascularization Endarterectomy versus Stenting Trial
SPACE	Stent-Protected Angioplasty versus Carotid Endarterectomy
ICSS	International Carotid Stenting Study
IL-6	Interleukin 6
DUS	Duplex ultrasound
PSV	Peak systolic velocity
ICA	Internal carotid artery
CCA	Common carotid artery
BMT	Best medical therapy
DAPT	Dual antiplatelet therapy
LDL	Low-density lipoprotein
ESVS	European Society for Vascular Surgery
TCAR	Transcarotid artery revascularization
Redo-CEA	Redo-endarterectomy
PTFE	Polytetrafluoroethylene
ISR	In-stent restenosis
POBA	Plain old balloon angioplasty
DCB	Drug-coated balloon
CBA	Cutting balloon angioplasty
